# Epithelial-mesenchymal cell competition coordinates fate transitions across tissue compartments during lung development and fibrosis

**DOI:** 10.21203/rs.3.rs-6189965/v1

**Published:** 2025-05-02

**Authors:** Kylie Klinkhammer, Rachel Warren, Joseph Knopp, Toan Nguyen, Stijn P. De Langhe

**Affiliations:** 1 Department of Medicine, Division of Pulmonary and Critical Medicine, Mayo Clinic, Rochester, MN 55905, USA.; 2 Department of Biochemistry and Molecular Biology, Mayo Clinic, Rochester, MN 55905, USA.

## Abstract

Morphogenesis and cell state transitions must be coordinated in time and space to produce a functional tissue. In this study, we reveal that lung mesenchymal Yap levels and fitness antagonize epithelial Yap levels and stemness during lung development and repair following bleomycin injury. Elevated mesenchymal Yap signaling and fitness antagonize epithelial Yap levels and stemness, accelerating alveolar epithelial differentiation while impairing branching during lung development or bronchiolization after bleomycin injury. Conversely, mesenchymal Snail/Slug sequesters Yap/Taz to direct an adipogenic differentiation program towards alveolar fibroblast 1 (AF1) during both lung development and the resolution of pulmonary fibrosis. On the other hand, Yap/Myc-Tead binding instructs a myogenic differentiation program. Through our experiments and modeling, we identify tissue-scale mechanical cooperation as a pivotal factor in orchestrating organ formation and regeneration.

## Introduction

The human lung’s intricate structure optimizes gas exchange through its extensive branching network of airways, culminating in 700 million alveoli^1^. Spanning an impressive 70 square meters, this network forms during the branching morphogenesis phase of development, where mesenchymal cells encircle the epithelial monolayer^2^. This crosstalk between epithelial and mesenchymal cells drives branching and differentiation processes. Interactions between these cell populations, along with their matrix, regulate the branching program and transition to the alveolar differentiation phase^3-5^. This epithelial-mesenchymal crosstalk remains essential during adult homeostasis and repair following injury^2,6,7^.

Early tissue recombination studies revealed that the mesenchyme plays a crucial role in instructing the epithelium, imparting its regional identity^8^. This instructive function becomes particularly evident in the injured adult lung. Following injury, developmental programs are reactivated to repair tissue damage and restore the organ to homeostasis. This paradigm applies to the lung, involving various signaling pathways such as Fgf, Hippo, and Wnt, which are either upregulated or downregulated in response to lung injury^9-11^. These signals form the foundation for communication and crosstalk between different cell types, both within the epithelium and between epithelial and mesenchymal cells.

In the adult lung, cartilage, airway smooth muscle, alveolar fibroblast 1 (AF1), and pericytes establish various mesenchymal stem cell niches. These niches are essential for maintaining and/or activating adult epithelial stem cells during homeostasis or initiating repair after injury^6,9,10,12-14^. Any disruptions in these interactions during lung development, homeostasis, or repair after injury can lead to the development of pulmonary diseases such as bronchopulmonary dysplasia (BPD), emphysema, and pulmonary fibrosis.

Fibrotic diseases, a major contributor to global morbidity and mortality, remain poorly understood. These diseases can affect various organs, ultimately leading to organ failure^15^. Fibrosis is believed to account for up to 45% of global deaths, with chronic lower respiratory fibrosis being a leading cause of death in the United States^15,16^. Idiopathic pulmonary fibrosis (IPF), a prevalent form of interstitial lung disease (ILD), results in alveolar remodeling and progressive loss of pulmonary function, respiratory failure, and death within five years of diagnosis^17,18^. IPF pathogenesis involves fibrotic remodeling, inflammation, and the loss of lung architecture^19^. Despite the underlying causes being elusive, genetic and experimental evidence supports the notion that chronic alveolar epithelial injury and inadequate repair of the respiratory epithelium are intrinsic to IPF disease pathogenesis. Histologically, respiratory epithelial cells in the lung parenchyma express atypical proximal airway epithelial and indeterminate cell type markers^20,21^, including goblet and basal cell (BC) markers, which are typically restricted to conducting airways. Fibrotic lesions and honeycomb structures replace alveoli, which are normally lined by alveolar type 1 (AT1) and AT2 cells. The bronchiolization process that leads to honeycomb structures is driven by intra-epithelial cell competition, where distal airway stem cells with high Yap/Myc levels become supercompetitors and replace the alveolar epithelium with bronchial epithelium^22^. Some abnormal airway/alveolar epithelial cells attempting to aid in regeneration persist in Krt8+ transitional states, which express genes associated with pro-fibrotic phenotypes^23-26^.

## Results

### Yap/Taz bind to Snail1/2 to drive alveolar fibroblast 1 differentiation during lung development.

The pulmonary mesenchyme includes multiple distinct cell lineages with various functions in lung development and in the pathogenesis and progression of debilitating respiratory conditions like idiopathic pulmonary fibrosis (IPF)^27,28^. Lung mesenchymal lineages are increasingly recognized as highly heterogeneous, including alveolar fibroblast 1 (AF1) and AF2, myofibroblasts, airway and vascular smooth muscle cells, pericytes and mesothelial cells. However, little is still known about how these different lineages arise during lung development and are regulated during lung fibrosis and resolution^29^. The AF1 lineage which forms the niche for alveolar type 2 (AT2) stem cells in the adult lung arises around E16.5 during lung development. During lung fibrosis AF1s give rise to myofibroblasts in response to TGFβ signaling and either undergo apoptosis or revert back to AF1s during fibrosis resolution^30-32^. Additionally, AF1 to myofibroblast differentiation in pulmonary fibrosis is thought to be mediated by Yap/Taz signaling^33,34^.

However, the role of Hippo signaling during mesenchymal lineage commitment during lung development remains largely unknown. To investigate this, we genetically inactivated Yap/Taz (Yap1^f/f^;Wwtr1^f/f^) or the Hippo kinases Mst1/2 (Stk4^f/f^;Stk3^f/f^) from the undifferentiated lung mesenchyme using *Tbx4-rtTa;Tet-Cre* starting from E9.5. Subsequently, we performed combined single nuclear RNAseq and ATACseq on E15.5 and E18.5 lungs. For each sample, we sequenced between 5,200 and 8,200 cells.

**Figure 1:**
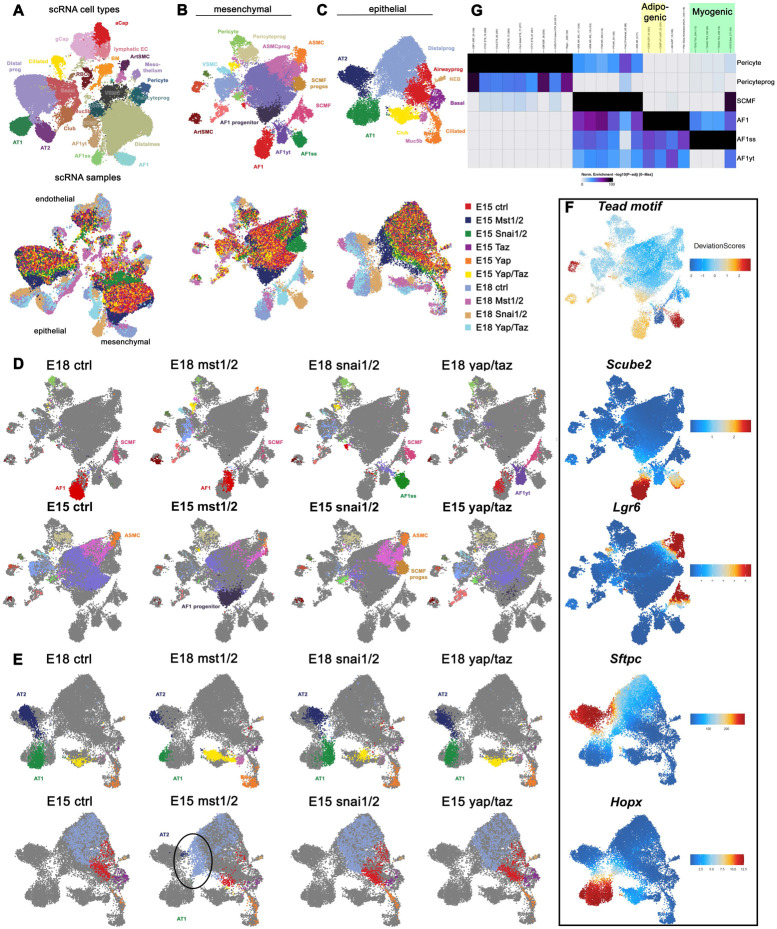
Single nuclei combined RNA and ATAC analysis of E15.5 and 18.5 mouse lungs. **(A)** Graph-based clustering and cell-type or sample annotation of integrated datasets from E15 and E18 ctrl and mutant lungs based on enriched gene expression and chromatin accessibility profiles by using ArchR. AF1ss (AF1 population in Snai1/2 inactivated mesenchyme), AF1yt (AF1 population in Yap/Taz inactivated mesenchyme). **(B,C)** Subsetting and clustering of **(B)** mesenchyme and **(C)** epithelium. **(D,E)** Graph-based clustering and cell-type annotation on individual samples of **(D)** mesenchyme and **(E)** epithelium. **(F)** Tead motif accessibility and representative gene expression for main mesenchymal and epithelial clusters. **(G)** Heatmap of differential transcription factor motif accessibility showing which regulatory factors are predicted to most active in each cell type. AF1ss (AF1 population in Snai1/2 inactivated mesenchyme) contains enrichment for myogenic differentiation and Tead transcription factors (green), AF1 is enriched for adipogenic factors (yellow), AF1yt (AF1 population in Yap/Taz inactivated mesenchyme) lacks Tead binding activity.

**Figure 2:**
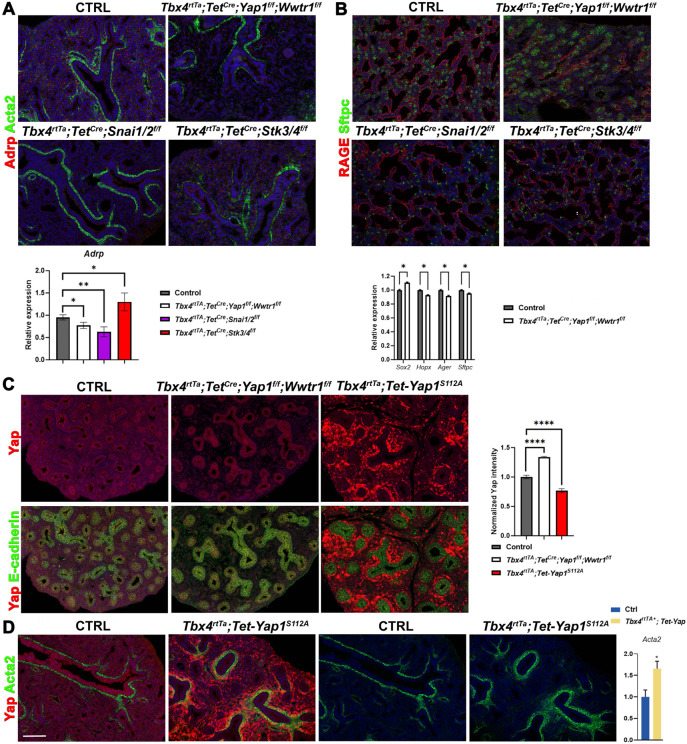
Snail1/2 and Yap/Taz are required for AF1 differentiation. **(A)** Immunostaining for Adrp and Acta2 on E15.5 control (n=4), *Tbx4-rtTa;Tet-Cre;Yap1^f/f^;Wwtr1^f/f^* (n=3), *Tbx4-rtTa;Tet-Cre;Snai1/2^f/f^* (n=3), and *Tbx4-rtTa;Tet-Cre;Stk3/4^f/f^* (n=2) lungs. Quantification of Adrp expression from images represented. **(B)** Co-immunostaining for Rage and Sftpc on E18.5 control, *Tbx4-rtTA;Tet-Cre;Yap1^f/f^*;*Wwtr1^f/f^, Tbx4-rtTA;Tet-Cre;Snai1/2^f/f^* and *Tbx4-rtTA;Tet-Cre;Stk3/4^f/f^* lungs doxycycline induced from E9.5. Nanostring nCounter RNA profiling on control (n=19) and *Tbx4-rtTA;Tet-Cre;Yap1*^f/f^;*Wwtr1*^f/f^ (n=21) lungs at E18.5. **(C)** Co-immunostaining for Yap and E-cadherin on E15.5 control (n=6), *Tbx4-rtTA;Tet-Cre;Yap1^f/f^*;*Wwtr1^f/f^* (n=3) and *Tbx4-rtTA;Tet-Yap^S112A^* (n=5) lungs doxycycline induced from E9.5. Scale bar 50μm. Quantification of epithelial Yap intensity on represented images. **(D)** Co-immunostaining for Yap and Acta2 on E15.5 control (n=5) and *Tbx4-rtTA;Tet-Yap^S112A^* (n=5) lungs doxycycline induced from E9.5. Scale bar 100μm. Quantification of average pixel intensity of Acta2 staining on whole lung represented images.

To our surprise, we found that inactivating *Yap1/Wwtr1* in the lung mesenchyme from E9.5 onwards impairs AF1 differentiation (marked by Scube2), which was confirmed by immunostaining for Adrp a well-known marker for AF1s (Fig.1A-E, Fig.2A). Conversely, inactivating *Stk3/4* in the lung mesenchyme from E9.5 onwards accelerates AF1 differentiation (Fig.1A-E). These findings suggest that mesenchymal Yap/Taz signaling plays a pivotal role in driving AF1 lineage commitment. This finding is intriguing because upon bleomycin injury, increased mesenchymal Yap/Taz-Tead signaling is believed to promote AF1 to myofibroblast differentiation^33,34^.

We were intrigued to explore whether Yap/Taz signaling in AF1 differentiation is partially mediated by a different transcriptional cofactor besides Teads. Snail1 and Snail2, which are highly expressed in AF1s and undifferentiated distal mesenchyme (Fig.S1A-E), have been demonstrated to bind Yap and Taz in bone marrow-derived mesenchymal stem cells (MSCs) to regulate their self-renewal and differentiation^35,36^. In these MSCs, Snail1 and Snail2 form binary complexes with Yap/Taz that not only control Yap/Taz protein levels but also regulate the expression of specific Yap/Taz-target genes^35,36^. To investigate a potential role for Snail1/2 in regulating mesenchymal lineage commitment during lung development, we added E15.5 and E18.5 *Tbx4-rtTA;Tet-Cre;Snai1/2^f/f^* lung samples, induced from E9.5, to our combined single nuclear RNAseq and ATACseq analysis.

We discovered that mesenchymal Snail1/2 binds to Yap/Taz to orchestrate an adipogenic differentiation program towards AF1. This is evident from reduced chromatin accessibility of CEBP binding motifs in lung AF1s from *Tbx4-rtTA;Tet-Cre;Snai1/2^f/f^* and *Tbx4-rtTA;Tet-Cre;Yap1^f/f^;Wwtr1^f/f^* mice (Fig.1G,Fig.S1F). Notably, the absence of Snail1/2 increases chromatin accessibility of Tead binding motifs, suggesting that Snail1/2 inhibits Yap/Taz-mediated Tead signaling (Fig.1G,F,Fig.S1F). A closer examination of Snail1/2 knockout mesenchyme compared to control mesenchyme reveals that the loss of Snail1/2 promotes mesenchymal differentiation along a myogenic differentiation program towards a myofibroblast lineage. This lineage exhibits increased chromatin accessibility of Stat1,Stat3, and Stat4^37-40^ as well as myogenic regulator factors including MyoD, Myf5, and MyoG^41-43^ binding motifs (Fig.1G,Fig.S1F).

### Mesenchymal Yap antagonizes epithelial Yap to coordinate fate transitions across tissue compartments.

We discovered that accelerated AF1 differentiation in the mesenchymal Stk3/4 knockout lungs is accompanied by accelerated alveolar epithelial differentiation (Fig.1E). Conversely, impaired AF1 differentiation in the mesenchymal Yap/Taz knockout lungs hinders the transition from a branching program to an alveolar differentiation program^3-5^ (Fig.2B), which occurs around E16.5 in parallel with AF1 differentiation. Notably, increased epithelial Yap signaling during lung development also impairs this transition^44^, implying that mesenchymal Yap levels may antagonize epithelial Yap levels. Indeed, we observe increased Yap levels in the epithelium of *Tbx4-rtTA;Tet-Cre;Yap1^f/f^;Wwtr1^f/f^* lungs but decreased epithelial Yap levels in lungs in which we overexpress a dominant, active version of the Hippo transcriptional effector Yap1^S112A^ in the lung mesenchyme (Fig.2C). This expression also activates a robust myogenic differentiation program, leading to increased smooth muscle cell differentiation (Fig.2D). Together, our findings indicate that lung mesenchymal Yap levels antagonize epithelial Yap levels during lung development to coordinate airway versus alveolar epithelial differentiation.

**Figure 3:**
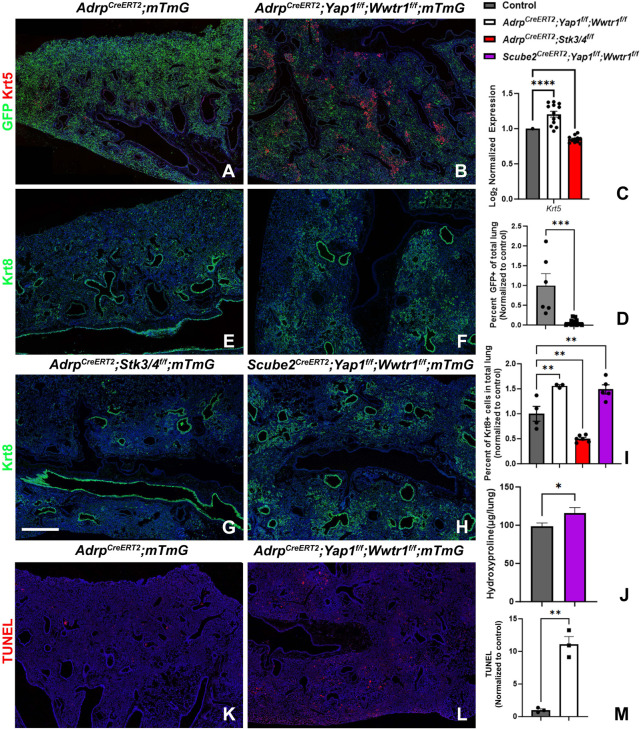
Loss of Yap/Taz in AF1s promotes bronchiolization after bleomycin injury. **(A-D)** Immunostaining for GFP and Krt5 on **(A)** control (n=7), **(B)** *Adrp-CreERT2; Yap1^f/f^;Wwtr1^f/f^;mTmG* (n=12) lungs 6 weeks after bleomycin injury. **(C)** Nanostring nCounter RNA analysis for Krt5 on control (n=42), *Adrp-CreERT2; Yap1^f/f^;Wwtr1^f/f^;mTmG* (n=13) and *Adrp-CreERT2;Stk3/4^f/f^* (n=11). **(D)** Image quantification of GFP in images represented in **(A,B). (E-H)** Immunostaining for Krt8 in **(E)** control, **(F)** *Adrp-CreERT2;Yap1^f/f^;Wwtr1^f/f^*, **(G)** *Adrp-CreERT2;Stk3/4^f/f^*, and **(H)** *Scube2-CreERT2;Yap1^f/f^;Wwtr1^f/f^* lungs 6 weeks after bleomycin injury. Scale bar 500μm. **(I)** Image quantification of Krt8 in images represented in **(E-H). (J)** Hydroxyproline analysis on bleomycin injured *Scube2-CreERT2;mTmG* controls (n=43) and *Scube2-CreERT2;Yap1^f/f^;Wwtr1^f/^;mTmG* (n=30). **(K-M)** TUNEL staining and quantification on bleomycin injured control *Adrp^CreERT2^;mTmG* (n=3) and *Adrp^CreERT2^;Yap1^f/f^;Wwtr1^f/f^;mTmG* (n=3) lungs.

The transition from a branching program to an alveolar differentiation program^3-5^ during lung development bears a striking resemblance to the binary decision made by distal airway stem cells in response to bleomycin injury. This decision involves either generating more airway epithelium, leading to bronchiolization, or alveolar epithelium. Notably, bronchiolization, a hallmark of pulmonary fibrosis, is mediated by an increase in airway epithelial Yap/Myc signaling^22^. Since mesenchymal Yap levels antagonize epithelial Yap levels during lung development, thereby coordinating airway versus alveolar epithelial differentiation, we hypothesized that mesenchymal Yap levels could also antagonize bronchiolization in pulmonary fibrosis. To investigate this, we induced bleomycin injury in adult lungs in which we inactivated Yap/Taz in AF1 cells using three distinct Cre lines: *Adrp-CreERT2, Tbx4-rtTa;Tet-Cre, and Scube2-CreERT2*, while simultaneously lineage tagging them with *Rosa26-mTmG*. Surprisingly, all three mouse models exhibited a striking increase in bronchiolization 6 weeks after bleomycin injury. This was characterized by an increase in aberrant basaloid (Krt8) and/or basal cells (Krt5) and increased pulmonary fibrosis based on hydroxyproline content compared to control lungs (Fig. 3A-J). Interestingly, we observed that *Adrp-CreERT2;Yap1^f/f^;Wwtr1^f/f^;mTmG* lungs exhibited fewer GFP-positive AF1s or myofibroblasts after bleomycin injury compared to *Adrp-CreERT2;mTmG* lungs, implying that inactivating Yap/Taz affects the fitness of AF1s. We found that *Yap/Taz* null AF1s become outcompeted by the bronchial epithelium and undergo apoptosis (Fig. 3A-M). Furthermore, since AF1s form the niche for AT2 cells, we observed that this loss of AF1s also leads to AT2 cell death. In contrast, the opposite phenotype was observed in *Adrp-CreERT2;Stk3/4^f/f^* lungs, where we inactivated the Hippo kinases *Mst1/2* in AF1 cells. These lungs exhibited decreased bronchiolization 6 weeks after bleomycin injury and no AF1 loss (Fig. 3C,G,I). We therefore propose that bronchiolization in pulmonary fibrosis also involves an epithelial-mesenchymal cell competition based on Yap levels between distal airway stem cells and AF1/myofibroblasts.

**Figure 4:**
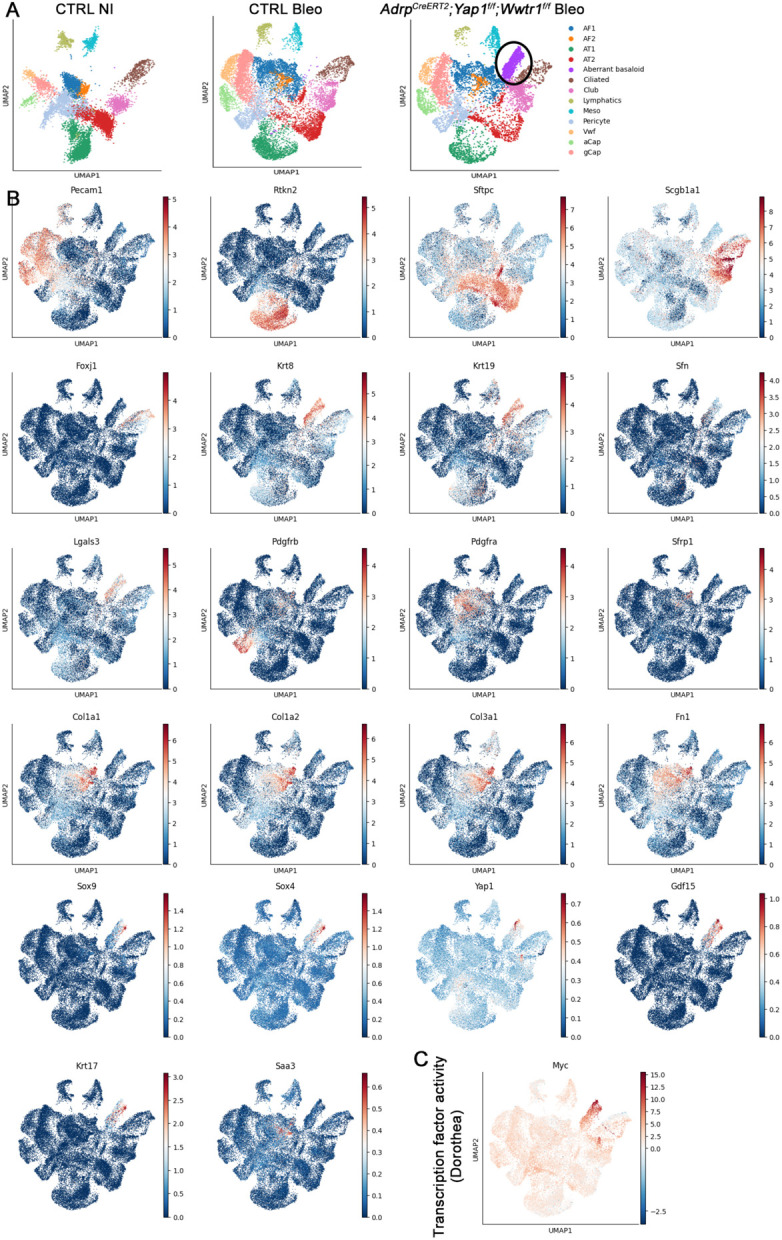
Loss of Yap/Taz in AF1s promotes the generation of aberrant basaloid cells after bleomycin injury. **(A)** clusters from scRNA-seq on control not injured (NI), bleomycin injured control, and bleomycin injured *Adrp-CreERT2;Yap1^f/f^;Wwtr1^f/f^*. Aberrant basaloid cell cluster is circled. **(B)** Gene expression of marker genes. **(C)** Myc transcription factor activity.

Next, we performed single-cell RNAseq on non-injured control and bleomycin-injured control lungs, as well as bleomycin injured *Adrp-CreERT2;Yap1^f/f^;Wwtr1^f/f^* lungs. Notably, only *Adrp-CreERT2;Yap1^f/f^;Wwtr1^f/f^* lungs exhibited a prominent aberrant basaloid cell population derived from the airway epithelium. This population displayed increased expression of *Krt5, Krt17, Yap1*, and *Gdf15* (Fig.4A,B) and increased inferred Myc transcriptional activity (Fig.4C). This aberrant basaloid phenotype bears striking resemblance to that observed in IPF^22-26,45^. Surprisingly, at 6 weeks post bleomycin injury, we found no difference between the AF1 populations from control versus *Adrp-CreERT2;Yap1^f/f^;Wwtr1^f/f^* injured lungs. This suggests that increased pulmonary fibrosis is a result of the rise in aberrant basaloid cells. The lack of significant transcriptional differences in AF1 populations from control versus *Adrp-CreERT2;Yap1^f/f^;Wwtr1^f/f^* injured lungs at 6 weeks post injury could be because most mutant AF1s have already been outcompeted at this time point.

**Figure 5:**
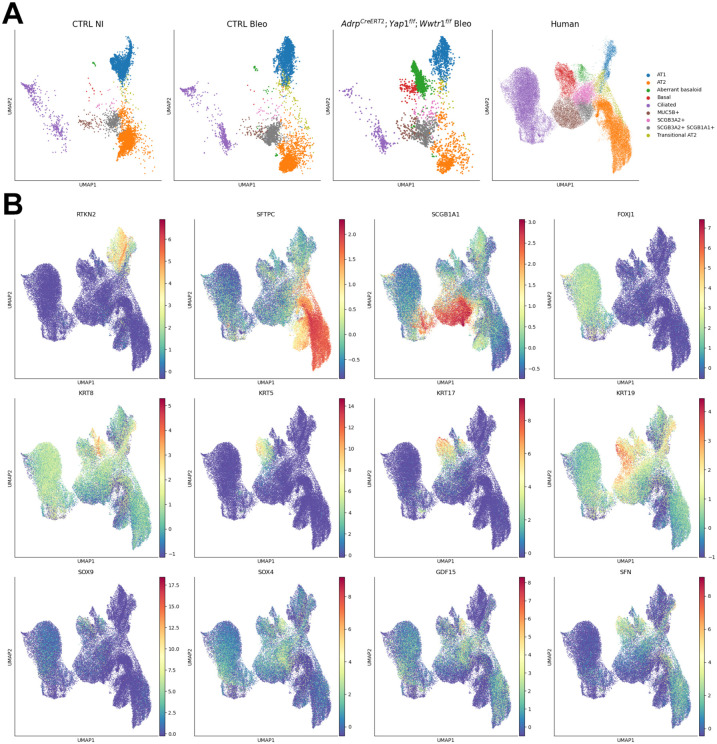
Loss of Yap/Taz in AF1s promotes the generation of aberrant basaloid cells after bleomycin injury similar to aberrant basaloid cells in IPF. **(A)** clusters from scRNA-seq on control not injured (NI), bleomycin injured control, bleomycin injured *Adrp-CreERT2;Yap1^f/f^;Wwtr1^f/f^* and human IPF. **(B)** Gene expression of marker genes.

To compare the aberrant basaloid populations observed in *Adrp-CreERT2;Yap1^f/f^;Wwtr1^f/f^* lungs after bleomycin injury with those observed in human IPF we integrated our mouse data sets with a human data set^26^ containing aberrant basaloid cells and found that both cell populations cluster together indicative of their similarity (Fig.5).

### Alveolar fibroblast 1 fitness levels are determined by their Myc levels.

**Figure 6:**
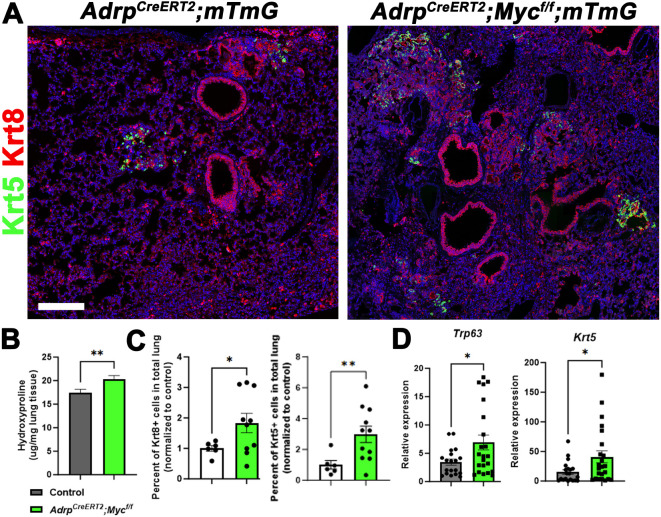
Loss of Myc in AF1s promotes bronchiolization after bleomycin injury. **(A)** Immunostaining for Krt8 and Krt5 on control (n=6) and *Adrp-CreERT2; Myc^f/f^;mTmG* (n=11) lungs 6 weeks after bleomycin injury. Scale bar 200μm. **(B)** Hydroxyproline analysis on bleomycin injured *AdrpCreERT2;mTmG* (n=23) and *Adrp-CreERT2;Myc^f/f^;mTmG* (n=34) lungs. **(C)** Image quantification of Krt8 and Krt5 in images represented in **(A).** **(D)** qPCR analysis on RNA from bleomycin injured *Cre-* controls (n=19) and *Adrp-CreERT2;Myc^f/f^;mTmG* (n=22) lungs for *Krt5* and *Tp63* genes.

Yap and Myc coordinately regulate genes required for cell proliferation, where activation of Myc leads to extensive association with its genomic targets, most of which are prebound by TEAD^46^. At these loci, recruitment of Yap is thought to be Myc-dependent and required for full transcriptional activation. This cooperation between Yap and Myc is thought to be critical for cell cycle entry and organ growth^46^. Since we have recently demonstrated that Myc and Yap cooperate in the lung epithelium to drive cell competition^22^ we wondered whether inactivation of *Myc* in AF1s prior to bleomycin injury would also render them vulnerable to becoming outcompeted by bronchial epithelium. Indeed we observe increased bronchiolization based on Krt8 and Krt5 expression (Fig.6A,C,D) and increased pulmonary fibrosis based on hydroxyproline content (Fig.6B) in *Adrp-CreERT2;Myc^f/f^;mTmG* mice 6 weeks after bleomycin injury.

### Myofibroblast to alveolar fibroblast 1 dedifferentiation requires a Yap/Taz/Snail1/2 transcriptional program.

**Figure 7:**
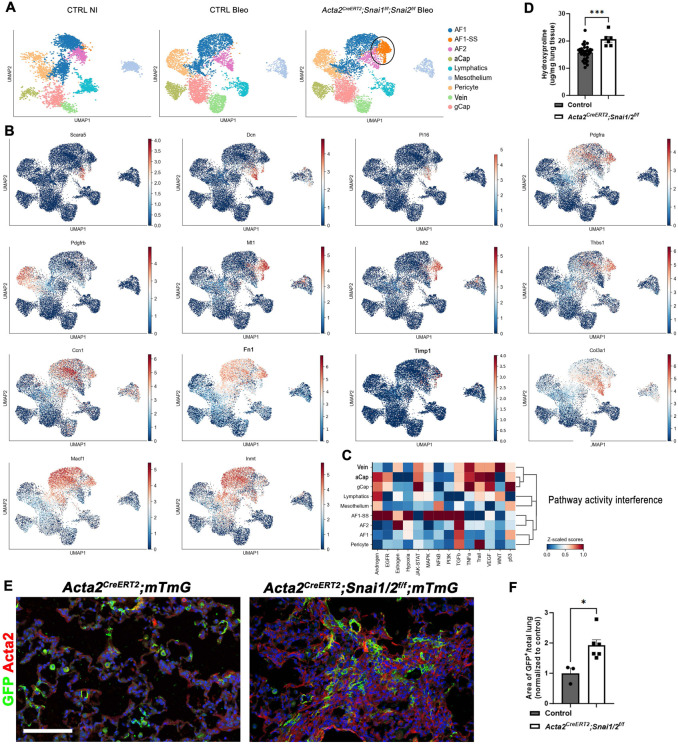
Snail1/2 are required for myofibroblast dedifferentiation into AF1s during the resolution of pulmonary fibrosis. **(A)** Mesenchymal cell clusters from scRNAseq on non injured control and bleomycin injured control and *Acta2-CreERT2;Snai1/2^f/f^* lungs tamoxifen induced 2 weeks after bleomycin injury. **(B)** Cell specific gene expression. **(C)** Fibrosis promoting pathway activities are upregulated in AF1-SS (AF1 population in Snai1/2 inactivated mesenchyme). **(D)** Hydroxyproline analysis of soluble collagen on control (n=30) and *Acta2-CreERT2;Snai1/2^f/f^*(n=7). **(E)** Co-immunostaining for GFP and Acta2 on bleomycin injured control (n=3) and *Acta2-CreERT2;Snai1/2^f/f^* (n=7) lungs tamoxifen induced 2 weeks after bleomycin injury. Scale bar 100 μm. **(F)** Quantification of GFP in images represented in **(E).**

Finally, we wondered if Snail1 and Snail2 are also crucial for the dedifferentiation of myofibroblasts into AF1s during the resolution of bleomycin-induced pulmonary fibrosis. To investigate this, we inactivated *Snail* and *Snai2* in myofibroblasts using *Acta2-CreERT2;Snai1/2^f/f^;mTmG* mice, starting 2 weeks after bleomycin injury and harvesting the lungs 4 weeks later (Fig.7). Single-cell RNA sequencing analysis of these lungs and lineage tracing of myofibroblasts revealed that inactivating *Snai1/2* from myofibroblasts after bleomycin injury hinders their dedifferentiation into AF1s and/or impairs their clearance through apoptosis. This is demonstrated by an increase in lineage labeled AF1s/Myofibroblasts (Fig7.E-F) and a cluster of myofibroblast-like cells (AF1-SS) with elevated expression of fibrosis-related genes (myofibroblast) population, including *Mt1, Mt2*, and *Thbs1*^47^ in our single cell analysis (Fig7.A-B). Pathway activity interference analysis indicates that different signaling pathways are active in myofibroblasts lacking Snail1/2 (AF1-SS) compared to normal AF1s, including Tgfβ and Hypoxia pathways (Fig.7C). Consequently, we observed increased pulmonary fibrosis 6 weeks after bleomycin injury, as measured by hydroxyproline content (Fig.7D).

## Discussion

The mechanical properties of the lung, such as tension or stiffness, are regulated to maintain the lung’s shape, which supports organ function. These bulk features originate from the mechanical properties of individual cells, which are primarily determined by actomyosin-dependent contractility and intercellular adhesion. Dynamic regulation of these molecular processes allows cells within tissues to exert forces on their neighbors, facilitating topological changes. During regeneration, this machinery also serves as a means to sense the growth rates of neighboring cells, ensuring uniform tissue growth across a population of cells with varying proliferation rates.

As cell density increases, neighboring cells exert increasing pressure on each other. This pressure activates mechanotransduction pathways in neighboring cells, prompting them to adjust growth by eliminating cells in overly crowded regions that disrupt homeostasis^48^. This process is also known as mechanical cell competition. Similar to the classic definition of cell competition, mechanical cell competition ultimately selects for cellular proliferation rate and favors the survival of cells that proliferate more rapidly. The vulnerability of any given cell to ‘sense’ a faster-proliferating neighbor depends on its susceptibility to compaction, which is ultimately determined by its mechanical properties relative to those of its neighbors. For instance, cells infected with bacterial pathogens exhibit altered mechanical properties compared to their uninfected neighbors. Uninfected cells are stiffer, enabling them to surround and compress the softer infected cells, eventually pushing them out of the tissue and limiting further propagation of infection^49^.

If softer, more compressible cells are not removed from the tissue, their increased persistence in the tissue would eventually affect the overall mechanical properties of the entire tissue and disrupt tissue morphology. Similarly, an ongoing, unbalanced presence of cells that are excessively stiff also poses a challenge in maintaining tissue organization. Cell-intrinsic mechanisms that control single-cell mechanics are crucial determinants of cellular fitness during homeostasis because they collectively impact bulk mechanical properties and overall tissue form. As such, forces must be balanced between neighboring cells and across the entire tissue to ensure that tissue morphology, and thus function, can be maintained. Hippo/yes-associated protein (Yap1) levels and its nuclear localization functions as a molecular sensor for cell density, but it also plays a role in maintaining tissue force balance^50^.

Yap1, a co-transcriptional activator, translocates to the nucleus in response to increased cellular tension. There, it induces TEAD-dependent gene expression, enabling the connection between mechanical stimuli and transcriptional responses, ultimately influencing downstream fate decisions^51,52^. While Yap activity varies among individual cells, the collective pattern of Yap localization across the entire tissue reflects its mechanical properties, which remain stable during homeostasis. Yap signaling serves as a fitness sensor, potentially acting as a molecular determinant of fitness. Previous studies have demonstrated that relative differences in Hippo/YAP signaling can drive cell competition in various scenarios^53-56^. Active shifts in the population heterogeneity of Yap signaling impact fate decisions^57,58^. Therefore, the heterogeneous patterns of Yap localization in adult tissues must be tightly regulated and selectively chosen at the individual cell level. Failure to do so could disrupt tissue form and function.

Our data suggest that inactivating Yap/Taz in alveolar fibroblast 1 cells (AF1s) and reducing their fitness before bleomycin injury disrupts the overall mechanical properties of the lung. This perturbation allows distal airway stem cells to increase their Yap levels and initiate bronchiolization of the lung parenchyma. We recently observed a similar response to injury when we inactivated Yap/Taz in AT2 stem cells, reducing their fitness^59^. These findings collectively reveal that different lung cell populations engage in a competitive process to regenerate or remodel the lung, aligning with the classical cell competition model initially identified in *Drosophila*^60,61^.

We further identify tissue-scale mechanical cooperation as a crucial factor in orchestrating organ formation and regeneration. This cooperation involves mesenchymal and epithelial cell populations sensing Yap or fitness levels in neighboring cells. We find that during lung development and repair after bleomycin injury, lung mesenchymal Yap levels and fitness antagonize epithelial Yap levels and stemness. Our data therefore suggest that tissues strive to restore tissue-wide homeostatic Yap levels as quickly as possible following injury. However, trying to maintain tissue-level function at all costs may lead to aberrant remodeling when there is a great disturbance in the force such as when millions of cells die off because of catastrophic injury.

Our findings that epithelial and mesenchymal populations sense each other’s Yap levels provide new insights into how the Hippo pathway drives regeneration and regulates organ size. Moving forward, future studies aiming to better characterize contractile heterogeneity and explore the tissue-level mechanisms that coordinate force balance across tissues during homeostasis and repair after injury will be important to find a cure for pulmonary fibrosis.

## Material and methods

### CONTACT FOR REAGENT AND RESOURCE SHARING

Further information and requests for resources and reagents should be directed to and will be fulfilled by the Lead Contact, Stijn De Langhe: delanghe.stijn@mayo.edu

### Experimental model and subject details

Both male and female mice were used in all experiments. Mice were bred in a pathogen-free environment on a standard laboratory setting of a 12h light, 12h dark exposure to mimic the day-night cycle. Room temperature was kept between 21°C and 24°C. *Stk3/4^f/f^* (RRID:IMSR_JAX:017635), *Yap1^f/f^*;Wwtr1*^f/f^* (RRID:IMSR_JAX:030532), *Tet-Yap1-H2BGFP* (RRID:IMSR_JAX:031279), *Adrp^CreERT2^* (kind gift from Dr. Ahlbrecht^62^), *Acta2^CreERT2^* (kind gift from Dr. Chambon^63^), *Scube2^CreERT2^* (kind gift from Dr. Sheppard^31^), *Rosa26-mTmG* (RRID:IMSR_JAX:007676), *Rosa26-Confetti* (RRID:IMSR_JAX:017492), *Snai1^f/f^;Snai2^f/f^* (RRID:IMSR_JAX:010686; RRID:IMSR_JAX:037485 ), *Tet^Cre^* (RRID:IMSR_JAX:006234), *Tbx4^rtTA^* (kind gift from Dr. Shi^64^).

Bleomycin mice were intratracheally instilled with 50μL bleomycin (0.8–2 U/kg body weight optimized for each strain, batch of bleomycin, and gender) as adults, 8 to 12 weeks old. For tamoxifen induction, *Acta2^CreERT2^, Adrp^CreERT2^, Scube2^CreERT2^* were placed on tamoxifen chow (rodent diet with 400 mg/kg tamoxifen citrate; Harlan Teklad TD.130860). *Adrp^CreERT2^* were placed on tamoxifen chow for 10 days at 2-3 months of age and were intratracheally instilled with bleomycin 4 weeks after being returned to normal rodent chow. *Acta2^CreERT2^* received bleomycin at 2-3 months of age and were put on tamoxifen chow 3 weeks later for 3 weeks until euthanasia. *Scube2^CreERT2^* mice were placed on tamoxifen chow at 2 months old for 3 weeks with an additional intraperitoneal tamoxifen injection (0.20 mg/g body weight, Millipore Sigma) on the last day of tamoxifen citrate feed, and after 3 weeks on normal laboratory chow, they were injured with bleomycin. *Tbx4^rtTA^*;*Tet^Cre^* mice were put on doxycycline containing chow (rodent diet with 625 mg/kg doxycycline; Harlan Teklad TD.09761) at weaning and remained on doxycycline chow until euthanasia and were intratracheally instilled with bleomycin at 2-3 months of age on doxycycline. All injured mice were euthanized 6 weeks post bleomycin injury. Dams of embryonic mice were put on doxycycline at embryonic day 9.5 (E9.5), and embryos were dissected at E13.5, E15.5, and E18.5. All experiments were approved by the Mayo Clinic Institutional Animal Care and Use Committee.

### Immunohistochemistry and Fluorescence

All staining was done on paraffin sections of formalin-fixed lungs. Immunofluorescent staining was performed with the following primary antibodies: chicken anti-GFP (1:500; GFP-1020; RRID:AB_10000240; Aves Labs Inc.), rabbit anti-Keratin 5 (1:200; clone EP1601Y; MA5-14473; RRID:AB_10979451; Thermo Fisher Scientific), rabbit anti-SFTPC (1:200; WRAB-9337; RRID:AB_2335890; Seven hills bioreagents), oat anti-RAGE (1:500; AF1145; RRID:AB_354628; R&D Systems), rat anti-Keratin 8 (1:100; TROMA-I; RRID:AB_531826; Developmental Studies Hybridoma Bank), mouse anti-alpha actin (smooth muscle actin (SMA), Acta2; 1:500; Clone 1A4; sc-32251; RRID:AB_262054; Santa Cruz Biotechnology Inc.), rabbit anti-Yap (1:100; 4912; RRID:AB_2218911; Cell Signaling), rabbit anti-ADRP (1:200, ab52356, RRID:AB_2223599, Abcam), mouse anti-Cadherin (E Monoclonal) (1:200, Clone 36, 610181, RRID:AB_397580, BD Biosciences), rabbit anti-Snail2 (1:100, Clone C19G7, 9585, RRID:AB_2239535). TUNEL assays were performed on paraffin sections of formalin-fixed lungs using the *In-Situ* Cell Detection Kit, TMR Red (12156792910, Roche).

After deparaffinization, slides were rehydrated through a series of decreasing ethanol concentrations, antigen unmasked by either microwaving in citrate-based antigen unmasking solution (Vector Labs, H-3300) or by incubating sections with proteinase K (7.5μg/ml) (Invitrogen, 25530-049) for 7 min at 37°C. Tissue sections were then washed in TBS with 0.1% Tween-20 and blocked with 3% Bovine Serum Albumin (BSA), 0.4% Triton X-100 in TBS for 30 min at room temperature followed by overnight incubation of primary antibodies diluted in 3% BSA, 0.1% Triton X-100 in TBS. The next day, slides were washed in TBS with 0.1% Tween-20 and incubated with secondary antibodies diluted in 3% BSA, 0.1% Triton X-100 in TBS for 3h at room temperature. All fluorescent staining was performed with appropriate secondary antibodies from Jackson Immunoresearch. Slides were mounted using Vectashield (Vector Labs, H-1000).

### Microscopy and imaging

Tissue was imaged using a micrometer slide calibrated Zeiss LSM800 Laser scanning confocal microscope using ZEN imaging software or Leica Stellaris 5 confocal microscope with LASX imaging software. Lungs were imaged using tiled stitched 20x images covering the entire cross-section of the left or lower right lung lobe from ≥6 different lungs. Representative images were chosen. Images were processed and analyzed using Zen blue (Zeiss), LASX (Leica) and Adobe Photoshop 2024 (Adobe) software.

### Image quantification

The total area of Krt5, Krt8 or GFP was determined using ImageJ (NIH) and analyzed based on the total lung area. Image quantification and analysis was performed in a double blinded fashion. Each quantification was ≥3 different mouse lungs.

### Quantitative real-time PCR

Total mRNA was extracted from lung accessory lobes stored in RNALater (Invitrogen, AM7021) and using Total RNA Kit I (Omega Biotek, R6834-02) according to the manufacturer’s instructions. RNA concentration was determined by spectrophotometry. cDNA was generated using Maxima^™^ First Strand cDNA Synthesis (Fisher Scientific, FERK1642) according to the manufacturer’s instructions. Gene expression was analyzed by quantitative RT-PCR using Taqman Gene Expression Assays (Applied Biosystems, 4369016) directed against the mouse targets *β-glucuronidase* (Mm00446953_m1), *Krt5* (Mm01305291_g1), *Trp63* (Mm00495788_m1). Quantitative real-time PCR was performed using a StepOne Plus system (Applied Biosystems). Data were presented as 2^−ΔΔCt^ with *β-glucuronidase* as the internal sample control normalized to control group. Each experiment was repeated with samples obtained from ≥3 different lung preparations.

### Nanostring

RNA was isolated from lung accessory lobes as described above. 100ng of RNA was hybridized with a custom RNA probe panel designed by NanoString (NanoString Technologies; DL_1206_C9662) for 16h according to manufacturer’s instructions. The RNA-probe hybridization was loaded on a NanoString cartridge and processed in a NanoString nCounter. Data was analyzed with Rosalind.bio (Rosalind, Inc) and Log2 Fold Changes were calculated and graphed. Each experiment was repeated with samples obtained from ≥3 different lung preparations.

### Hydroxyproline Assay.

The right lobes were flash frozen in dry ice at the time of harvest and stored at −80°C. For acid hydrolysis, the lobes were baked in a 70°C oven without lids for 2 days until completely dry. The weights of dry lobes were measured and 500μl of 6N HCl were added to each sample. The lungs were then hydrolyzed in an 85°C oven for 2 days with occasional vortexing. The hydrolysates were cooled at room temperature and centrifuged at maximum speed for 10 min. The supernatants then were transferred to fresh 1.5 mL tubes and centrifuged at maximum speed for 10 min. Each sample or standard was diluted with citrate-acetate buffer (5% citric acid, 1.2% glacial acetic acid, 7.24% sodium acetate, and 3.4% sodium hydroxide) in a 96-well plate. Chloramine-T solution (1.4% chloramine-T, 10% N-propanol, and 80% citrate-acetate buffer) was added, and the mixture was incubated for 20 min at room temperature. Then, Ehrlich’s solution (1.27M p-dimethylaminobenzaldehyde, 70% N-propanol, 20% perchloric acid) was added to each sample and the samples were incubated at 65°C for 20 min. Absorbance was measured at 550 nm. Standard curves were generated for each experiment using reagent hydroxyproline (Sigma H-1637) as a standard. The amount (μg) of hydroxyproline were calculated by comparison to the standard curve.

### 10x Genomics Single Cell Multiome ATAC+ Gene Expression Sequencing

Embryonic day E15.5 or E18.5 lungs were collected and flash-frozen in a dry ice/ethanol slurry. Frozen tissues were homogenized with a dounce homogenizer and nuclei were isolated according to 10 x Genomics Nuclei Isolation from Embryonic Mouse Brain for Single Cell Multiome ATAC+ Gene Expression Sequencing. Nuclei viability was assessed and total nuclei were counted. ATAC and gene expression libraries were then constructed from the nuclei according to the 10 x Genomics Chromium Next GEM Single Cell Multiome ATAC+ Gene Expression User Guide. Sequencing was performed on a NextSeq 500. Alignment was performed using the ‘Cellranger count’ function provided in 10 x Genomics single-cell gene expression software. For secondary analysis we used the R-package ArchR to apply standard quality control metrics and unsupervised clustering for the generation of initial UMAP projections and integrate multiomics data.

### 10x Genomics Single Cell Fixed RNA Profiling (Flex) Analysis

Cells from 2 50μm formalin fixed paraffin embedded (FFPE) tissue sections were deparaffinized and manually dissociated with a pellet pestle using 1.0mg/mL Liberase TH in RPMI according to manufacturer’s directions (10x Genomics).Cells were counted using Countess II FL Automated Cell Counter (Thermo Fisher Scientific) and up to 10,000 cells per sample were hybridized with mouse WTA Probes (10x Genomics; PN-2000703-2000718) for 17h according to manufacturer’s instructions. Post-hybridization cells were counted and pooled with equal number of cells per sample for a target recovery of 128,000 cells. GEM generation and final library preparations and sequencing were completed by the Mayo Genomics Research Core according to manufacturer’s instructions on an Illumina NextSeq. Fastq files were analyzed with Cell Ranger Pipeline using 10x Genomics Cloud Analysis.

### In situ for Snai1 and Snai2

Whole-mount in situ hybridization was performed as previously described^65^ with antisense probes against full length Snai1 or Snai2.

### Statistics and Reproducibility.

All results are expressed as mean values ± SEM. The ‘n’ represents biological replicates and can be found in the figure legends. The significance of differences between 2 sample means was determined by two-tailed unpaired *t*-test (assuming unequal or equal variances as determined by the F-test of equality of variances). All datasets followed a normal distribution and P values less than 0.05 were considered statistically significant. The number of samples to be used was based on the number of experimental paradigms multiplied by the number in each group that is necessary to yield statistically significant results (based on power analysis, to reject the null hypothesis with 80% power (type I error = 0.05).

## Supplementary Material

Supplement 1 Figure S1 Loss of Snail1/2 promotes a myogenic differentiation program.
**(A,B)** *In situ* hybridization for *Snai1* and *Snai2* on E13.5 mouse lungs.
**(C-E)** Co-immunostaining for Snail2 and Acta2 on E15.5 control and *Tbx4-rtTA;Tet-Cre;Snai1/2^f/f^* lungs doxycycline induced from E9.5. Scale bar 100μm.
**(F)** Heatmap of differential transcription factor motif accessibility showing which regulatory factors are predicted to most active in each cell type. AF1ss (AF1 population in Snai1/2 inactivated mesenchyme) contains enrichment for myogenic differentiation and Tead transcription factors (green) while AF1 is enriched for adipogenic factors (yellow).
**(G)** Graph-based clustering and cell-type or sample annotation of integrated mesenchymal datasets from E15 and E18 ctrl and *Tbx4-rtTa;Tet-Cre;Snai1/2^f/f^* lungs based on enriched gene expression and chromatin accessibility profiles by using ArchR. Representative gene expression for SCMF, Pericyte. AF1 and AF1ss mesenchymal clusters.
