# Supplementary figures and images for: Epithelial-mesenchymal cell competition coordinates fate transitions across tissue compartments during lung development and fibrosis
Source: Res Sq. 2025 May 2:rs.3.rs-6189965. Preprint. [Version 1] doi: 10.21203/rs.3.rs-6189965/v1 (PMC12060972; doi:10.21203/rs.3.rs-6189965/v1)

Figure S1

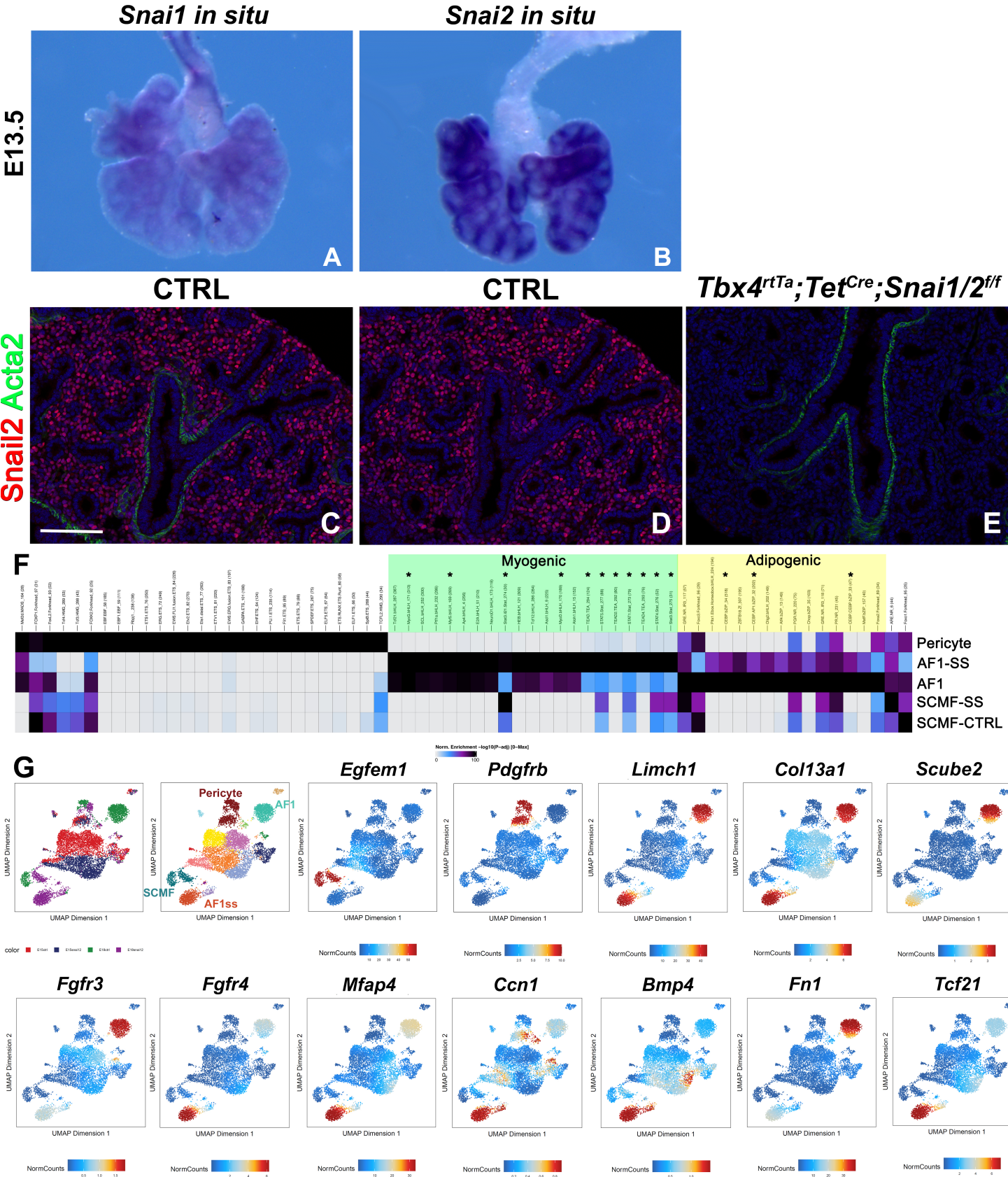

Supplement: Supplement 1 — Figure S1 Loss of Snail1/2 promotes a myogenic differentiation program. (A,B) In situ hybridization for Snai1 and Snai2 on E13.5 mouse lungs. (C-E) Co-immunostaining for Snail2 and Acta2 on E15.5 control and Tbx4-rtTA;Tet-Cre;Snai1/2f/f lungs doxycycline induced from E9.5. Scale bar 100μm. (F) Heatmap of differential transcription factor motif accessibility showing which regulatory factors are predicted to most active in each cell type. AF1ss (AF1 population in Snai1/2 inactivated mesenchyme) contains enrichment for myogenic differentiation and Tead transcription factors (green) while AF1 is enriched for adipogenic factors (yellow). (G) Graph-based clustering and cell-type or sample annotation of integrated mesenchymal datasets from E15 and E18 ctrl and Tbx4-rtTa;Tet-Cre;Snai1/2f/f lungs based on enriched gene expression and chromatin accessibility profiles by using ArchR. Representative gene expression for SCMF, Pericyte. AF1 and AF1ss mesenchymal clusters. [file NIHPPrs6189965v1-supplement-1.pdf]
